# Oxidative Damage and Antioxidant Defense in Ferroptosis

**DOI:** 10.3389/fcell.2020.586578

**Published:** 2020-09-17

**Authors:** Feimei Kuang, Jiao Liu, Daolin Tang, Rui Kang

**Affiliations:** ^1^The Third Affiliated Hospital, Guangzhou Medical University, Guangzhou, China; ^2^Department of Surgery, UT Southwestern Medical Center, Dallas, TX, United States

**Keywords:** ferroptosis, cell death, ROS, antioxidant, redox

## Abstract

Many new types of regulated cell death have been recently implicated in human health and disease. These regulated cell deaths have different morphological, genetic, biochemical, and functional hallmarks. Ferroptosis was originally described as a carcinogenic RAS-dependent non-apoptotic cell death, and is now defined as a type of regulated necrosis characterized by iron accumulation, lipid peroxidation, and the release of damage-associated molecular patterns (DAMPs). Multiple oxidative and antioxidant systems, acting together autophagy machinery, shape the process of lipid peroxidation during ferroptosis. In particular, the production of reactive oxygen species (ROS) that depends on the activity of nicotinamide adenine dinucleotide phosphate (NADPH) oxidases (NOXs) and the mitochondrial respiratory chain promotes lipid peroxidation by lipoxygenase (ALOX) or cytochrome P450 reductase (POR). In contrast, the glutathione (GSH), coenzyme Q10 (CoQ10), and tetrahydrobiopterin (BH_4_) system limits oxidative damage during ferroptosis. These antioxidant processes are further transcriptionally regulated by nuclear factor, erythroid 2-like 2 (NFE2L2/NRF2), whereas membrane repair during ferroptotic damage requires the activation of endosomal sorting complexes required for transport (ESCRT)-III. A further understanding of the process and function of ferroptosis may provide precise treatment strategies for disease.

## Introduction

**FIGURE 1:**
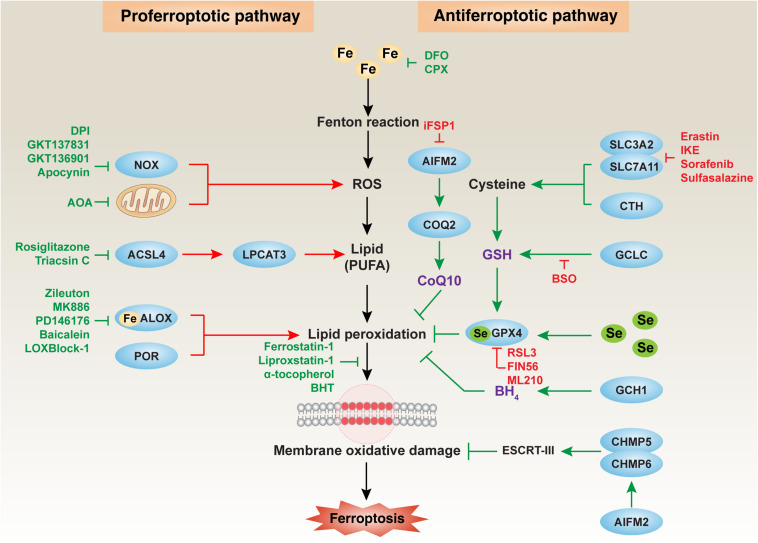
The major mechanism of oxidative damage and antioxidant defense in ferroptosis. Ferroptosis is an iron-dependent oxidative cell death caused by ROS from the Fenton reaction and subsequent lipid peroxidation. Multiple oxidative and antioxidant systems control the process of membrane oxidative damage during ferroptosis. In particular, NOX-dependent and mitochondrial respiratory chain-dependent ROS production promotes lipid peroxidation by ALOX or POR. The production of lipids, especially PUFA, requires the activation of the ACSL4-LPCAT3 pathway. In contrast, the GSH, CoQ10, and BH_4_ systems limit oxidative damage during ferroptosis. Membrane repair during ferroptotic damage requires the activation of the ESCRT-III pathway. ACSL4, acyl-CoA synthetase long-chain family member 4; AIFM2, apoptosis-inducing factor mitochondria-associated 2; ALOX, lipoxygenase; BH_4_, tetrahydrobiopterin; CHMP5, charged multivesicular body protein 5; CHMP6, charged multivesicular body protein 6; COQ2, coenzyme Q2, polyprenyltransferase; CoQ10, coenzyme Q10; CTH, cystathionine gamma-lyase; ESCRT-III, endosomal sorting complexes required for transport-III; GCH1, GTP cyclohydrolase 1; GCLC, glutamate-cysteine ligase catalytic subunit; GPX4, glutathione peroxidase 4; GSH, glutathione; LPCAT3, lysophosphatidylcholine acyltransferase 3; NOX, nicotinamide adenine dinucleotide phosphate (NADPH) oxidase; POR, cytochrome P450 oxidoreductase; ROS, reactive oxygen species; Se, selenium; SLC3A2, solute carrier family 3 member 2; SLC7A11, solute carrier family 7 member 11.

The survival and death of cells are strictly controlled by various signals and molecules (Fulda et al., 2010). Physiological cell death is essential for normal function and tissue development. But pathological cell death may have side effects that cause inflammation and threaten our health. The first classification of cell death was based on morphological criteria proposed by pathologists in the 1970s. The pathologists suggested that cell death has three main forms, namely apoptosis (type I), autophagy (type II), and necrosis (type III) (Schweichel and Merker, 1973). The typical morphological changes of apoptosis are nuclear chromatin concentration and the formation of apoptotic bodies in the cytoplasm (Elmore, 2007). This is different from cell swelling and membrane rupture in necrosis (Golstein and Kroemer, 2007) and the formation of cytoplasmic double membrane vesicles in autophagy (a lysosome-dependent degradation process) (Xie et al., 2015). The latest cell death classification was formulated and is recommended by the Cell Death Nomenclature Committee. Generally, cell death is divided into accidental cell death and regulated cell death (Galluzzi et al., 2018). Accidental cell death is a passive process, whereas regulated cell death is an active process that plays an important role in the pathogenesis of the disease (Galluzzi et al., 2018). In the past 20 years, many new types of regulated cell death (e.g., necroptosis, pyroptosis, ferroptosis, entotic cell death, netotic cell death, parthanatos, lysosome-dependent cell death, autophagy-dependent cell death, alkaliptosis, and oxeiptosis) have been identified in various models (Tang et al., 2019). Although they may share several common signals (e.g., redox signals), different forms of regulated cell death require special molecular machinery to trigger cell death (Tang et al., 2019). In this review, we summarize the major ways in which oxidative stress and antioxidant defense regulate ferroptosis, which is a form of iron-dependent cell death driven by lipid peroxidation (Figure 1).

## The Basic Properties of Ferroptosis

The concept of ferroptosis is derived from precision medicine for tumors that targeted RAS mutation signals (Dolma et al., 2003; Yang and Stockwell, 2008). RAS is a proto-oncogene and is frequently mutated in human cancer, leading to tumorgenesis and therapy resistance. Drug screening identified that the small molecular compounds erastin and RSL3 can selectively kill RAS-mutant cancer cells, but not RAS wild-type cells (Dolma et al., 2003; Yang and Stockwell, 2008). Later, it was proved that the anticancer activity of erastin and RSL3 depends on the induction of a new type of iron-dependent cell death, termed ferroptosis (Dixon et al., 2012). Although initial research showed that ferroptosis may be different from the classical cell death (e.g., apoptosis, necrosis, and autophagy-dependent cell death) (Dixon et al., 2012), recent studies demonstrate that there is a close relationship between ferroptosis, necrosis, and autophagy (Chen M.S. et al., 2017; Muller et al., 2017; Li et al., 2020; Lin et al., 2020; Liu et al., 2020c; Xie et al., 2020a). Ferroptotic cells usually exhibit cell membrane rupture and the release of intracellular contents, especially damage-associated molecular patterns (DAMPs) (Wen et al., 2019; Dai et al., 2020b), and are therefore classified as a type of regulated necrosis (Conrad et al., 2016). Increased autophagy, especially several types of selective autophagy [e.g., ferritinophagy (Hou et al., 2016), lipophagy (Bai et al., 2019), clockophagy (Yang M. et al., 2019), and chaperone-mediated autophagy (Wu et al., 2019)], promotes ferroptosis, indicating that ferroptosis is related to an abnormal intracellular degradation pathway.

Although the direct effectors of ferroptosis are unclear, iron accumulation and lipid peroxidation seem to play a central role in regulating the process of ferroptosis (Dixon et al., 2012; Yang et al., 2016; Shintoku et al., 2017; Wenzel et al., 2017; Li et al., 2020). Iron is an essential nutrient for cell proliferation, but iron overload can cause iron toxicity and lead to cell damage, even death. The balance of iron in cells and in the body is controlled by an integrated system. Pathways for abnormal iron metabolism, such as increasing the iron absorption and reducing iron storage or iron output, may cause ferroptosis through at least two ways. One is iron-mediated reactive oxygen species (ROS) production through the Fenton reaction (Dixon et al., 2012). The other is involved in the activation of iron-containing enzymes, such as lipoxygenase (ALOX) (Yang et al., 2016; Shintoku et al., 2017; Wenzel et al., 2017; Li et al., 2020). Finally, the accumulation of iron causes lipid peroxidation, which is the process of oxidative degradation in lipids [especially polyunsaturated fatty acids (PUFAs)] (Yuan et al., 2016; Doll et al., 2017; Kagan et al., 2017), leading to subsequent membrane damage and rupture. In contrast, an increased membrane repair ability through the activation of charged multivesicular body protein 5 (CHMP5) and charged multivesicular body protein 6 (CHMP6), which then mediate the endosomal sorting complexes required for transport (ESCRT)-III pathway, limits ferroptosis (Dai et al., 2020c). Notably, multiple oxidative stress and antioxidant defense pathways are involved in shaping ferroptotic responses (discussed later). This process is further regulated by epigenetic, transcriptional, posttranscriptional, and posttranslational mechanisms (Dai et al., 2020a; Wu et al., 2020). In particular, the activation of nuclear factor, erythroid 2-like 2 (NFE2L2/NRF2) plays a major transcriptional regulatory role in the suppression of ferroptosis through the induction of expression of antioxidants or iron metabolism genes (Sun et al., 2016a,b). Functionally, impaired ferroptosis (due to its excessive activation or cellular defects) is increasingly recognized as the cause of human diseases, especially neurodegenerative diseases, cancer, and infectious diseases as well as tissue damage (Xie et al., 2016; Stockwell et al., 2017).

## Oxidative Damage in Ferroptosis

Oxidative damage results from an imbalance between the generation of free radicals and the body’s ability to neutralize or eliminate their harmful effects through antioxidants. ROS-mediated lipid peroxidation is the key step that drives ferroptosis. Below, we describe the main cellular sources of ROS and the main regulators of lipid peroxidation during ferroptosis (Figure 1).

### Mitochondria-Mediated ROS Production

Mitochondria play a key role in regulating cell energy and cell death signal transduction. In addition to producing adenosine triphosphate, mitochondria are also the main source of ROS production (Zorov et al., 2014). The production of mitochondrial ROS mainly occurs during the oxidative phosphorylation in the electron transport chain located on the inner membrane of the mitochondria. Electrons leak from complex I and complex III on the electron transport chain, resulting in a partial reduction of oxygen to form superoxide anion (O_2_^⋅–^). Subsequently, O_2_^⋅–^ is rapidly disproportionated into hydrogen peroxide (H_2_O_2_) by the superoxide dismutase 2 (SOD2) in the mitochondrial matrix and superoxide dismutase 1 (SOD1) in the intermembrane space. Overall, O_2_^⋅–^ and H_2_O_2_ produced during this process are called mitochondrial ROS, which is related to the loss of mitochondrial membrane potential (ΔΨm) (Zorov et al., 2014). The reduction of ΔΨm is a common sign of apoptosis and ferroptosis, but their regulatory mechanisms are different. In mitochondria, cytochrome C, somatic (CYCS) plays an essential role in generating ΔΨm. The translocation of CYCS from the mitochondria to the cytoplasm is an important event that initiates the loss of ΔΨm in the process of apoptosis induced by mitochondrial ROS (Yang et al., 1997). However, changes in CYCS position and the subsequent activation of apoptosis effector caspases are not observed during ferroptosis (Dixon et al., 2012), indicating that different mechanisms control ΔΨm during ferroptosis.

**FIGURE 2:**
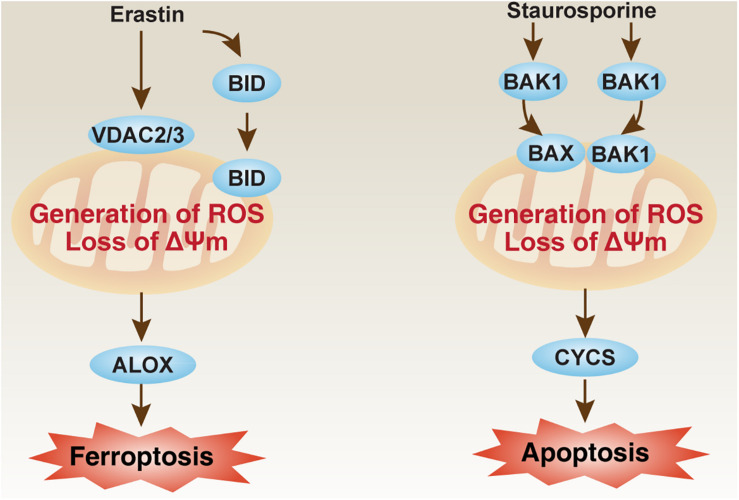
The role of mitochondria in ferroptosis and apoptosis. The loss of mitochondrial membrane potential (ΔΨm) and the production of ROS are implicated in ferroptosis and apoptosis by different pathways. Erastin can bind VDAC2 and VDAC3 to induce BID-dependent ferroptosis through ALOX, whereas BAK1 and BAX are required for staurosporine-induced apoptosis through CYCS. ALOX, lipoxygenase; BAK1/BAK, BCL2 antagonist/killer 1; BAX, BCL2-associated X, apoptosis regulator; BID, BH3 interacting domain death agonist; CYCS, cytochrome C, somatic; ROS, reactive oxygen species; VDAC, voltage-dependent anion channel.

Some important mitochondrial apoptosis regulators, such as those in the voltage-dependent anion channel (VDAC) family and BCL2 family, are also involved in the regulation of ferroptosis in a context-dependent manner. VDAC, also known as mitochondrial porin, is the most abundant protein in the outer mitochondrial membrane. In the VDAC family, VDAC2 and VDAC3 are considered to be directly targeted by erastin to induce ferroptosis (Figure 2; Yagoda et al., 2007). As a negative feedback mechanism, the proteasome degradation of VDAC2 and VDAC3 that depends on NEDD4 E3 ubiquitin protein ligase can block erastin-induced ferroptosis in melanoma cells (Yang et al., 2020b). NEDD4-like E3 ubiquitin protein ligase (NEDD4L) inhibits ferroptosis by degrading lactotransferrin protein (Wang et al., 2020). VDAC2 is also a direct target of lipid-derived electrophile-induced carbonylation proteins during ferroptosis caused by RSL3 (Chen et al., 2018). Therefore, posttranslational modulation of VDAC2 or VDAC3 may weaken or exacerbate ferroptosis sensitivity. In addition to VDAC, the BCL2 family is also an important regulator of mitochondrial outer membrane permeability, which consists of many members that promote or inhibit apoptosis (Youle and Strasser, 2008). Pro-apoptotic BCL2 family members, such as BCL2 antagonist/killer 1 (BAK1/BAK) and BCL2-associated X, apoptosis regulator (BAX), are required for staurosporine-induced apoptosis (Wei et al., 2001), but not required for erastin-induced ferroptosis in fibroblasts (Dixon et al., 2012; Figure 2). However, BH3 interacting domain death agonist (BID), a pro-apoptotic BCL2 family member, mediates mitochondrial ROS-induced ferroptosis in neuronal cells (Neitemeier et al., 2017; Jelinek et al., 2018). These findings increase the likelihood that apoptotic or ferroptotic death requires different types of changes in mitochondrial membrane, which are further regulated by different members of VDAC and BCL2 families.

In addition, mitochondrial energy sensors or mitochondrial quality control systems plays a dual role in ferroptosis. AMP-activated protein kinase (AMPK) is a key sensor of cellular energy and regulates ferroptosis through its phosphorylated substrate. AMPK-mediated BECN1 (a key autophagy regulator) phosphorylation promotes ferroptosis through the inhibition of system xc^–^ activity (Song et al., 2018). In contrast, AMPK-mediated acetyl-CoA carboxylase alpha (ACACA) phosphorylation blocks ferroptosis through the inhibition of fatty acid biosynthesis (Lee et al., 2020). Additional signals, currently unknown, are needed to explain the substrate selectivity of AMPK-related ferroptosis regulation. Although many types of selective autophagy promote ferroptosis, mitophagy (an important mitochondrial quality control system) may play a context-dependent role in ferroptosis. On the one hand, mitophagy can maintain a healthy number of mitochondria to promote survival against ferroptosis (Gao et al., 2019). On the other hand, excessive mitophagy may cause metabolic stress and subsequent production of mitochondrial ROS, leading to ferroptosis (Basit et al., 2017). The interaction of mitochondria and other organelles in ferroptosis remains to be explored.

### NOX-Mediated ROS Production

For a long time, the production of O_2_^⋅–^ by transmembrane nicotinamide adenine dinucleotide phosphate (NADPH) oxidase (NOX) has been regarded as an important function in professional phagocytes, such as macrophages and dendritic cells (Bedard and Krause, 2007). In addition to phagocytes, other cells also express NOX to produce O_2_^⋅–^ or H_2_O_2_ by transporting electrons across the membrane. The human genome encodes seven members of the NOX family, including five NOX proteins [NOX1, cytochrome B-245 beta chain (CYBB/NOX2), NOX3, NOX4, and NOX5] and two dual oxidases (DUOX1 and DUOX2). NOX-derived ROS plays a broad role in various physiological and pathological conditions (e.g., development, infection, immunity, and cell death) (Bedard and Krause, 2007). As an important regulator of lipid raft-derived redox signaling platforms, NOX participates in the induction of apoptosis (Jin et al., 2011). Similarly, NOX1-, CYBB-, and NOX4-mediated ROS production is also involved in the initiation of ferroptotic cancer cell death by inducing lipid peroxidation (Xie et al., 2017; Chen et al., 2019; Yang W.H. et al., 2019; Yang et al., 2020a), indicating a wide role for NOXs in cell death. In cancer cells, the activity of NOXs in ferroptosis is further affected by oncogenes and tumor suppressors. For example, the loss of tumor suppressor TP53 inhibits the accumulation of dipeptidyl-peptidase-4 (DPP4/CD26) in the nucleus, thereby increasing plasma-membrane associated DPP4-dependent lipid peroxidation and subsequent ferroptosis via the formation of the DPP4-NOX1 complex (Xie et al., 2017). During the activation of oncogenic RAS, NOX1 mediates the production of ROS (Adachi et al., 2008), which may promote ferroptosis through the activation of the extracellular signal-regulated kinase (ERK) pathway (Yagoda et al., 2007). More research is needed to clearly define how different NOX members, coupled with impaired genetic signals in tumors, cause ferroptosis.

### ALOX-Mediated Lipid Peroxidation

Reactive oxygen species-mediated lipid peroxidation is mainly accomplished by ALOX, which is a dioxygenase that contains non-heme iron. ALOX includes six members (ALOXE3, ALOX5, ALOX12, ALOX12B, ALOX15, and ALOX15B) and catalyzes the stereotactic insertion of oxygen into PUFAs, especially arachidonic acid (AA) and adrenic acid (AdA), in a tissue- or cell-dependent manner. For example, ALOX5, ALOXE3, ALOX15, or ALOX15B mediates ferroptosis caused by erastin or RSL3 in BJeLR, HT1080, or PANC1 cells (Yang et al., 2016; Shintoku et al., 2017; Wenzel et al., 2017; Li et al., 2020). ALOX15 or ALOX12 mediates TP53-induced ferroptosis in cancer cells following different stimuli (Ou et al., 2016; Chu et al., 2019). Phosphatidylethanolamine-binding protein 1 (PEBP1) can be used as an adaptor protein for ALOX15 and enhances the activity of ALOX15 in the induction of ferroptosis *in vitro* (Wenzel et al., 2017). However, ALOX12/15 may not be important for ferroptotic damage in mice caused by glutathione peroxidase 4 (GPX4) depletion in kidney (Friedmann Angeli et al., 2014) or T cells (Matsushita et al., 2015). Therefore, when evaluating the sensitivity of ferroptosis, it is necessary to first detect the basic expression levels of different ALOX members.

Lipoxygenase-mediated lipid peroxidation is firstly initiated by the generation of AA/AdA derivatives mediated by ACSL4 and LPCAT3 (Yuan et al., 2016; Doll et al., 2017; Kagan et al., 2017). ACSL4 catalyzes the combination of free AA/AdA and CoA to form AA/AdA-CoA derivatives and promotes their esterification to phospholipids, while LPCAT3 then catalyzes the biosynthesis of AA/AdA-CoA and membrane phosphatidylethanolamine (PE) to form AA/AdA-PE. ALOX then mediates the peroxidation of AA/AdA-PE to generate AA/AdA-PE-OOH (e.g., 5-HETE, 11-HETE, and 15-HETE, but not 12-HETE), which leads to membrane injury during ferroptosis. Therefore, the genetic or pharmacological inhibition of the ACSL4-LPCAT3-ALOX pathway inhibits ferroptosis *in vitro* and *in vivo*. Although ACSL4-independent ferroptosis may exist, increased ACSL4 expression is a biomarker of ferroptosis (Yuan et al., 2016).

### POR-Mediated Lipid Peroxidation

Cytochrome P450 reductase (POR) plays a major role in the metabolism of drugs and steroids. POR supplies electrons to microsomal cytochrome P450 from NADPH (Riddick et al., 2013). Consequently, the destruction of POR affects the activity of all microsomal P450 enzymes. In addition to ALOX-mediated lipid peroxidation, POR-mediated lipid peroxidation plays an alternative role in mediating ferroptosis. In particular, POR binds its cofactors, such as flavin mononucleotide (FMN) and flavin adenine dinucleotide (FAD). This complex mediated electron supplementation to cytochrome P450 from NADPH is required for erastin-, FIN56-, ML210-, or RSL3-induced lipid peroxidation and subsequent ferroptosis in melanoma and other cancer cells (Zou et al., 2020). Although the exact mechanism of POR-mediated lipid peroxidation is still unknown, POR may accelerate the cycling between ferrous and ferric iron in the heme component of cytochrome P450 (Zou et al., 2020). Given that the conditional knockout of POR in the liver leads to a decrease in the metabolism and lipid accumulation in mice (Henderson et al., 2003), the pathological role of POR-mediated ferroptosis in tissue damage and metabolism disease is worthy of further study.

## Antioxidant Defense in Ferroptosis

Cellular protection against oxidative damage in ferroptosis is organized at multiple levels. The synthesis of antioxidants, such as glutathione (GSH), coenzyme Q10 (CoQ10) and tetrahydrobiopterin (BH_4_), is the main defense strategy in the process of ferroptosis, which is related to multiple enzymes or proteins (Figure 1). In addition to the antioxidant systems discussed below, some antioxidant proteins, such as peroxiredoxins (PRDXs) (Lu et al., 2019; Qi et al., 2019; Lovatt et al., 2020) and thioredoxin (Llabani et al., 2019), can also block ferroptotic cell death. Therefore, an integrated antioxidant defense network exists in different cells.

### GSH System

Glutathione is an active tripeptide, formed by the condensation of glutamic acid, cysteine, and glycine. As an important antioxidant, glutathione is used to treat liver diseases, tumors, poisoning, cataracts, and aging diseases. The pharmacological inhibition of GSH synthesis and utilization is a classic method of inducing ferroptosis (Dixon et al., 2012; Yang et al., 2014). There are two main sources of cysteine production for GSH synthesis, namely the system xc^–^ pathway and the transsulfuration pathway (McBean, 2012; Lewerenz et al., 2013). System xc^–^ is a transmembrane sodium-independent and chloride-independent transporter of cystine and glutamic acid, which contains two key components [solute carrier family 7 member 11 (SLC7A11/xCT) and solute carrier family 3 member 2 (SLC3A2/CD98)] (Lewerenz et al., 2013). After being transported into the cell by system xc^–^, cystine is oxidized to cysteine, which is then used for glutamate-cysteine ligase catalytic subunit (GCLC/GCL)-mediated GSH synthesis. The inhibition of system xc^–^ (using erastin, sorafenib, and sulfasalazine) or GCL (using buthionine sulfoximine) triggers ferroptosis in various cells (Conrad and Pratt, 2019). The expression or activity of system xc^–^ is affected by epigenetics, transcription, and posttranscriptional and posttranslational regulators, such as TP53 (Jiang et al., 2015), NFE2L2 (Chen D. et al., 2017), BRCA1-associated protein 1 (BAP1) (Zhang Y. et al., 2018), mucin 1, cell surface-associated (MUC1) (Hasegawa et al., 2016), or BECN1 (Song et al., 2018), leading to complex feedback mechanisms to control GSH levels in ferroptosis. The transsulfuration pathway is a metabolic pathway that involves the interconversion of cysteine and homocysteine through intermediate cystathionine (McBean, 2012). Cystathionine gamma-lyase (CTH/CGL)-mediated decomposition of cystathionine is required for cysteine production. This process is inhibited by cysteinyl tRNA synthetase 1 (CARS1/CARS), an enzyme that charges tRNA^Cys^ with cysteine in the cytoplasm. In contrast, knocking down CARS1 increases resistance to ferroptosis by activating the transsulfuration pathway (Hayano et al., 2016).

The main anti-ferroptotic activity of GSH is related to GPX4, which reduces phospholipid hydroperoxide production (AA/AdA-PE-OOH) to AA/AdA-PE-OH (Yang et al., 2014). GPX4 is a selenium-containing protein whose activity is regulated by GSH. As an essential trace element, the function of selenium depends on a unique functional group, namely the selenol (-SeH) group. In the catalytic cycle of GPX4, active selenol (-SeH) is oxidized by peroxide to selenic acid (-SeOH), and then reduced by GSH to intermediate selenide disulfide (-Se-SG) (Ingold et al., 2018). GPX4 is further activated by the second GSH, releasing glutathione disulfide (GS-SG) (Ingold et al., 2018). GPX4 inhibitors (e.g., RSL3, ML162, ML210, FIN56, and FINO2) are also known as classic ferroptosis activators, although their activities and effects are still different (Conrad and Pratt, 2019). In addition to ferroptosis, GPX4 also mediates antioxidant defense in apoptosis (Ran et al., 2003), necroptosis (Canli et al., 2016), and pyroptosis (Kang et al., 2018), suggesting a context-dependent role of GPX4 in cell death.

### CoQ10 System

Coenzyme Q10 is a vitamin-like endogenously produced isoprenyl benzoquinone compound that occurs naturally in the human body and is highest in the heart, liver, kidney, and pancreas (Hernandez-Camacho et al., 2018). CoQ10 exists in oxidized form (ubiquinone) and reduced form (ubiquinol). The effective function of mitochondria depends on various cofactors, such as L-carnitine, α-lipoic acid, and CoQ10 (Pagano et al., 2014). CoQ10 is particularly interesting because it not only supports the mitochondrial respiratory chain, but also acts as a powerful antioxidant by neutralizing free radicals in various membrane structures (Teran et al., 2018). CoQ10 not only inhibits apoptosis (Chen et al., 2011), but also ferroptosis (Shimada et al., 2016). For example, the application of farnesyl pyrophosphate (an upstream product of CoQ10 synthesis) or idebenone (a hydrophilic analog of CoQ10) prevents ferroptosis caused by FIN56 (Shimada et al., 2016). In contrast, inhibiting the production of CoQ10 may accelerate ferroptotic cell death. In particular, apoptosis-inducing factor mitochondrial-related 2 (AIFM2/FSP1/AMID), a traditional regulator of apoptosis in the mitochondria (Wu et al., 2002), can mediate the production of CoQ10 to inhibit ferroptosis in a GSH-independent manner (Bersuker et al., 2019; Doll et al., 2019). This process requires the N-myristoylation of AIFM2, which results in the translocation of AIFM2 to the cell membrane (Bersuker et al., 2019; Doll et al., 2019). The depletion of CoQ10 biosynthesis enzyme [e.g., coenzyme Q2, polyprenyltransferase (COQ2)] may reverse the anti-ferroptotic activity of AIFM2 (Bersuker et al., 2019; Doll et al., 2019). Of note, the increased accumulation of AIFM2 in the cell membrane may also inhibit ferroptosis by activating CHMP5- and CHMP6-mediated ESCRT-III membrane repair mechanisms, which are independent of CoQ10 (Dai et al., 2020d). Statin drugs inhibit 3-hydroxy-3-methylglutaryl–coenzyme A reductase (HMG-CoA), a rate-limiting step that converts HMG-CoA to mevalonate in the production of cholesterol. Interestingly, statins can cause a decrease in CoQ10, thereby increasing the sensitivity of ferroptosis (Shimada et al., 2016). Further understanding of the antioxidant capacity of CoQ10 may provide benefits for reducing ferroptosis-related damage.

### BH_4_ System

Tetrahydrobiopterin is a natural nutrient that can be used as a cofactor for various enzymes, such as tryptophan hydroxylase, phenylalanine hydroxylase, tyrosine hydroxylase, nitric oxide (NO) synthase, and glyceryl ether mono-oxygenase (Werner et al., 2011). Functionally, BH_4_ is involved in the biosynthesis of some neurotransmitters, such as 5-hydroxytryptamine, dopamine, noradrenaline, adrenaline, and melatonin (Werner et al., 2011). Exogenous dopamine or melatonin has been shown to suppress erastin- or hemin-induced ferroptosis in various cells (NaveenKumar et al., 2019; Wang et al., 2016), but it remains unclear whether BH_4_-mediated endogenous dopamine or melatonin production regulates ferroptosis. In addition, BH_4_ plays a redox role in the catalysis of L-arginine, O_2_, and NADPH to form NO. The oxidation of BH_4_ to BH_2_ causes an uncoupling of NOS, thereby forming O_2_^⋅–^ instead of NO. O_2_^⋅–^ reacts rapidly with NO to form peroxynitrite, and nitrite can further uncouple NOS (Werner et al., 2011). The activation of the NO pathway is implicated in ferroptosis-related tissue injury (Deng et al., 2020). In particular, L-arginine induces acute pancreatitis in mice through the activation of ferroptosis-induced sterile inflammation, which is further regulated by circadian rhythms (Liu et al., 2020d). The synthesis and recycling of BH_4_ is a dynamic process, and GTP cyclohydrolase-1 (GCH1) is the rate-limiting enzyme for the biosynthesis of BH_4_. GCH1-mediated BH_4_ production prevents ferroptosis by inhibiting lipid peroxidation (Kraft et al., 2020), indicating that BH_4_ has antioxidant activity during cell death. Overall, these findings demonstrate the prosurvival role of BH_4_ in protecting against ferroptotic damage.

## Ferroptosis in Disease

**FIGURE 3:**
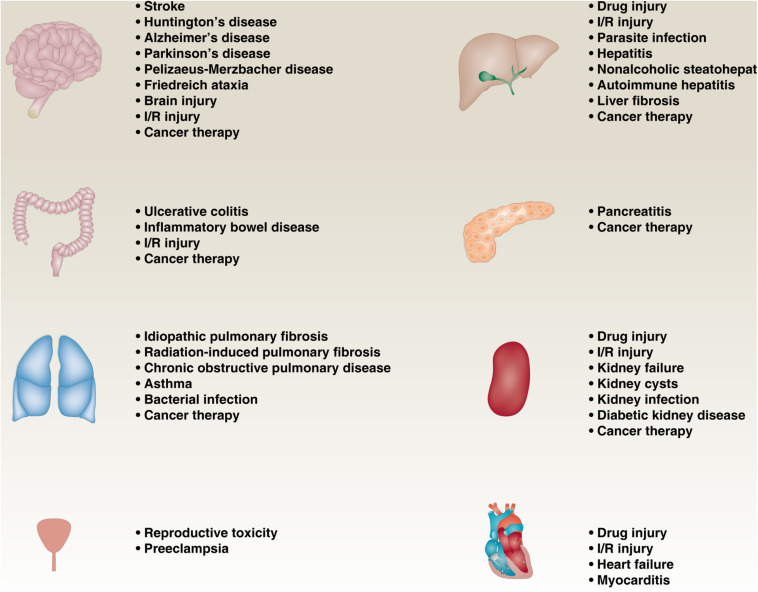
The role of ferroptosis in diseases. Impaired or excessive ferroptotic pathway is involved in various diseases, such as neurodegenerative diseases, infectious diseases, cancers, and ischemia-reperfusion (I/R) injury diseases.

More and more reports showing that impaired or excessive ferroptotic pathway in various diseases, such as neurodegenerative diseases, infectious diseases, cancers, and ischemia-reperfusion (I/R) injury diseases (Figure 3). In cancer pathology, ferroptosis not only inhibits tumor growth (Daher et al., 2019), but also promotes tumor formation (Dai et al., 2020b), depending on the type, stage, and microenvironment of the tumor. Iron-induced ferroptotic damage is implicated in Huntington’s disease (HD), Alzheimer’s disease (AD), and Parkinson’s disease (PD) (Skouta et al., 2014; Do Van et al., 2016; Zhang Y.H. et al., 2018; Hirata et al., 2019), although oxytosis has long been considered to be the main mode leading to neuronal cell damage caused by glutamate toxicity (Tan et al., 2001). Inflammation mediated by ferroptotic cell death can promote pancreatitis (Liu et al., 2020d), liver fibrosis (Tsurusaki et al., 2019; Zeng et al., 2020), chronic obstructive pulmonary disease (COPD) (Park et al., 2019; Wang and Tang, 2019; Yoshida et al., 2019), inflammatory bowel disease (Mayr et al., 2020), and preeclampsia (Zhang et al., 2020). In addition, inhibiting ferroptosis can prevent I/R damage to various tissues, especially liver, kidney, brain, and heart (Linkermann et al., 2014; Skouta et al., 2014; Martin-Sanchez et al., 2017; Muller et al., 2017; Fang et al., 2019). Therefore, the development of pharmacological agents that regulate ferroptosis under these pathological conditions is crucial.

## Conclusion and Perspectives

Ferroptosis was discovered not long ago, and there have been more and more studies related to it in recent years. This is because the core signals of ferroptosis (iron accumulation and lipid peroxidation) are often observed abnormally in various diseases and pathological conditions. Like other types of regulated cell death, ferroptosis may be caused by an imbalance between oxidation and antioxidant systems (Chen et al., 2020). In particular, NOX-dependent and mitochondrial respiratory chain-dependent ROS formation facilitates lipid peroxidation, whereas the GSH, CoQ10, and BH_4_ systems play a major role in limiting oxidative damage during ferroptosis. However, the process and function of ferroptosis needs to be explored. An unresolved central issue is that these oxidative damage, antioxidant defense mechanisms, and membrane repair mechanisms are also involved in the regulation of other kinds of non-ferroptotic cell death (Bae et al., 2011; Liu et al., 2020a). Thus, although they may share a common upstream mechanism, the identification of unique downstream effectors may distinguish ferroptosis from non-ferroptotic cell death. Similarly, it remains a challenge to distinguish the pathological role of ferroptosis and non-ferroptotic cell death in disease (Hotchkiss et al., 2009). In addition, the complexity of autophagy and lipid metabolism in the regulation of ferroptosis should be further clarified (Liu et al., 2020b; Xie et al., 2020b) and therefore provide a reasonable explanation for regulating ferroptosis in a context-dependent manner.

## Author Contributions

All authors conceptualized and wrote the manuscript.

## Conflict of Interest

The authors declare that the research was conducted in the absence of any commercial or financial relationships that could be construed as a potential conflict of interest.
